# Person-centred care in nursing homes during the COVID-19 pandemic: a cross sectional study based on nursing staff and first-line managers’ self-reported outcomes

**DOI:** 10.1186/s12912-023-01437-z

**Published:** 2023-08-21

**Authors:** Helen Lindner, Annica Kihlgren, Margaretha Norell Pejner

**Affiliations:** 1 School of Health Sciences SE, Faculty of Medicine and Health, Örebro University, 701 82 Örebro, Sweden; 2 Older People’s Health and Living Condition, Örebro University, Örebro, Sweden; 3 Department of Home Care, Halmstad Municipality, Halmstad, Sweden

**Keywords:** COVID-19, Nursing homes, Person-centred care, Self-reported outcomes, Leadership

## Abstract

**Background:**

COVID-19 has presented many difficulties in providing person-centred care (PCC) in nursing homes (NH). Factors such as organisational support, work condition and leadership may play a crucial role in supporting the performance of PCC during COVID restrictions. The study aim was to evaluate nursing staff and manager perceptions of the opportunities to perform person-centred care during the COVID-19 pandemic.

**Methods:**

Nursing staff (NS) (*n* = 463) and First Line Managers (FLM) (*n* = 8) within all NHs in one community filled in the SVENIS questionnaire which consists of five areas: perceived organizational support, work climate, person-centred care, work conditions and leadership. A Kruskal-Wallis test was used to perform inter-group comparisons and standard multiple regression was used to investigate which factor contributed most to perform PCC.

**Results:**

The comparison analyses indicate that staff from *nursing homes for persons with dementia* had the highest opportunities to perform PCC during the pandemic. The day shift staff had more opportunities to perform PCC than night shift staff. The results from the standard multiple regression show that a NA’s current nursing home was the most significant variable affecting the opportunities to perform PCC. The analyses of both the comparison analyses and the regression suggest that day shift staff from nursing homes for persons with dementia had the highest opportunities to perform PCC during the pandemic. The same group also rated the importance of leadership as high for performing PCC.

**Conclusion:**

Despite the COVID-19 restrictions and all the criticism directed against the care of older people; the day staff felt that they conducted PCC. Staff in nursing homes for dementia had the highest opportunities for PCC and this may be because they are better prepared to provide care for the individual in NH. The importance of leadership was also evident, which means that investment in FLMs is seen as necessary.

## Background

The COVID-19 pandemic triggered a global wave of fear and uncertainty unlike anything we have experienced in recent history. In March 2020, countries around the world implemented different drastic measures to slow the spread of the virus, such as lockdowns, stay-at home orders [1] and in Sweden, social distancing restrictions to protect older persons in nursing homes (NHs) [2]. On 1st April 2020, Swedish government issued an ordinance prohibiting external visits at NHs for older persons. Just overnight, older persons in NHs were prohibited to have physical contact with their loved ones. Their daily physical contact was restricted only to nursing staff (NS) and other residents [2–4]. The COVID restrictions imposed unforeseen challenges to perform person-centred care (PCC) for older people living in NHs [3, 4]. The question became “how do NS in NHs continue to practice PCC under such restrictive routines?”

In nursing care, PCC is an approach in which the patient is seen as an individual with unique needs, preferences, values and beliefs, and is actively involved in all aspects of his/her care. It focuses on enhancing quality of life and promoting physical, emotional and social well-being of the patient [5–9]. There are national and international consensuses describing the concept of PCC and its practice in elderly care [8, 9]. PCC has been associated with higher quality of life and quality of care for older persons in NHs [10–12]. Factors such as social engagement, meaningful activities, along with social contacts are essential for quality of life among older people in NHs [13, 14].

Achieving PCC requires a holistic perspective in which the individual is seen in his/her context, and it is based on two different perspectives; personal and organizational. The personal perspective concerns the meeting between individuals and what knowledge the NS have about the older person’s background, current situation, and future. NS provide direct care to the older persons living in NH [15, 16] using the PCC guidelines [17]. However, during the pandemic, the social distancing restrictions implied that the older persons were not allowed to meet their relatives, and this led to a feeling of social isolation. Furthermore, the NS performed COVID hygiene routines using face masks, gloves and aprons [9]. The fact that treatments and care were performed by NS wearing protective equipment and distancing restrictions between NS and the older person could also intensify this sense of isolation.

The organizational perspective of PCC concerns working methods and work climate [18]. It is about the opportunities and support available in the organisation to enable the practice of PCC. Leadership of nursing home managers play an essential role for the quality of home care [19] because it maintains the standards of the care being delivered and promoting optimal work condition [20–23]. During the pandemic, the first line managers (FLMs) of Swedish nursing homes were facing unforeseen organisational issues, such as a strained economy and an increased sick leave of NS (around 25% of the total workforce), which added enormous pressure on work condition. Furthermore, leadership is associated with PCC provision and facilitate work condition of NS to practice PCC. Under the COVID-related restrictions, it was even more crucial for the FLMs to maintain the practice of PCC in NHs.

Given the unusual circumstances relating to caring for the older persons during the pandemic, it would be valuable to study the extent to which staff felt it was possible to perform PCC during this time and to gain more information about their perceptions of working under these circumstances. Moreover, it would be insightful to investigate how factors regarding the NS, such as workplace, age, sex, education, year of employment, would predict the opportunities for PCC practice in NHs during the pandemic. The aim of the study was to evaluate nursing staff and first-line managers’ perceptions of the opportunities to perform person-centred care during the COVID-19 pandemic.

## Method

### Study design

A cross- sectional study was used to answer the study aim and the STROBE Statement—checklist of items for reports of observational studies was used to guide the steps.

### Setting

The study was carried out during year 2021 in all nine NHs and one short stay residence for older persons in one municipality in southwestern Sweden. There are 59 units and 24 of them are dementia care units with 192 apartments. The rest of the units are oriented towards somatic care, with a total of 384 apartments. Each apartment has a room with a kitchenette and a bathroom. The residents may furnish their apartment with their own furniture. A somatic care unit has 8–10 apartments and a dementia care unit has 6 to 8 apartments. The dementia care units have higher number of NS compared to the somatic care units. The NS in dementia care units were selected by the FLMs based on the staff specific interest in dementia care, although they do not formal training in dementia care. The NHs are staffed around the clock, but during the evening and night shift, the NS serve one or two units, instead of just the regular one.

### Participants

An inclusion criterion for participants in this study were NS and FLMs that have permanent positions in the NH and worked more than three days a week. We excluded NS that work hourly in the nursing home. The NS consist of Nurse Assistants (NAs) with a high school education in nursing for three years and Care Assistants (CAs) that could have a short diploma in nursing. Registered nurses (RNs) are stationed at a NH or work on consultant basis whereas occupational therapists (OT) work on consultant basis but do not station at a particular NH. The total eligible NS and FLMs were 711 and 18 respectively (*N* = 729 in total). Due to around 25% sick leave among the NS and silence decline for participation, a total of 463 NS and 8 FLMs participated in the study (*n* = 471 in total).

### Demographic variables and instrument

The following demographic variables were collected: sex, age, education, years of working with elderly care, years of working in the current nursing home, type of employment and the nursing home type.

 The Swedish National Inventory of Care and Health in Nursing Homes for the elderly (SVENIS) was used to collect data and it consists of five areas: perceived organizational support, work climate, person-centred care, work conditions and leadership.

#### Perceived organizational support - Swedish Demand–Control–Support Questionnaire (DCSQ)

The DCSQ was used to examine the perceived work situation. The instrument consists of three subscales with questions answered on a scale from 1 (= no, almost never) to 4 (= yes, often). The perceived demands subscale has 5 questions (scores 5–20), the perceived control subscale has 6 questions (scores 6–24), and the perceived support subscale has 6 questions (scores 6–24) [24]. The Cronbach’s alpha for the three sub-scales of psychological demands, decision latitude and social support were α = 0.78, 0.50, 0.82. One item is “we often discuss how we can provide care based on individual needs.” Another item is “ We have the freedom to change our work routine based on individual needs.”

#### Work climate - Person-Centred Climate Questionnaire (PCQ-S)

The PCQ-S was used to assess the extent to which staff perceive the residential environment in nursing homes to be person-centred. The instrument contains 14 statements about the psychosocial living environment and is answered on a Likert scale with scores ranging from 0 (= No, strongly disagree) to 5 (= Yes, strongly agree). Satisfactory psychometric properties have been reported for the Swedish version of the PCQ-S with good estimate of reliability Cronbach a = 0.88 and construct validity [25], One item is “I feel welcome in my workplace” and another item is “ I can be myself in my workplace”.

#### Person-centred care – Person-Centred Assessment Tool (P-CAT)

P- CAT was used to explore the extent to which nurses believe that the organization, processes and care provided on their ward reflect the key elements of person-centred care. The instrument comprises 13 statements describing a person-centred approach, answered on a Likert scale with scores ranging from 1 (= No, completely disagree) to 5 (= Yes, completely agree). The Swedish version of P- CAT has been shown to have satisfactory psychometric properties of reliability (Cronbach’s a = 0.75) and construct validity in Swedish Cronbach’s alpha 0.75 for the total scale [26, 27]. One item is “help to plan activities for the residents.” Another item is “talk to and be together with the residents.”

#### Work conditions - Person-Directed Care (PDC)

PDC includes questions about the extent to which staff feel they are able to work in a person-centred way. The survey contains 15 statements that describe a person-centred approach. Staff can indicate on a scale of 1 (= very rarely or never) to 5 (= very often or always) how often they can perform the step in the statements. The scale is psychometrically tested by the designer All constructs were conceptually distinct and internally consistent, and, as expected, all were positively correlated [28]. One item is “Do you have to finish your work tasks?”. Another item is “ are you allowed to learn new knowledge/skills at your workplace?”.

#### Leadership behavior questionnaire

Leadership assessed with 6 questions about the manager’s commitment to the individual employee’s work performance. The questions are answered on a scale ranging from 1 (= to a very low degree) to 5 (= to a very high degree). Average points are calculated for each question. Furthermore, 24 questions about the manager’s actions and attitudes are answered on a scale between 0 (= does not apply at all) and 5 (= fully applies). Higher scores indicate more positive leadership [29]. One item is “delegation of responsibilities.” Another item is “ discussion of new ideas.”

### Data collection

Due to the restriction that prevailed due to COVID-19 several meetings were held on TEAM and information was sent out by mail. The manager in charge and the chief nurse were invited to a meeting and informed about the study and to get their permission to conduct the study. The manager in charge subsequently informed the FLMs at all NHs with oral and written information about the study and then e-mailed the questionnaire to the FLMs who printed it out on paper. The FLM then informed the NS, with oral and written information, and distributed the questionnaire to their NS in May 2021. A reminder was sent out after one month. When the questionnaire was answered, at the latest by the end of July 2021, the questionnaire was returned by the NS in sealed envelopes to the last author.

### Ethical considerations

The study was approved by the Swedish Ethical Review Authority Dnr:2019–04463. Permission also was given by the developer of SVENIS for data collection (via email communication with the last author). Following the ethical procedure, voluntary participation was emphasized in the information and any decline of participation would not lead to negative consequences. The participants gave their consent by sending back the questionnaires in sealed envelopes to the last author.

In order to protect the confidentiality of the participants, every single returned questionnaire and each unit was coded with a number. The code lists were stored in two password-locked accounts in which only accessible by the last author. As stated in the information letter, all data were reported at group level and hence the risk of exposing a particular NH or a group of participants was considered as minimal.

### Statistical analysis

The analysis began with investigating the internal consistency of the SVENIS questionnaire, using the data from all five scales, even though the study did not aim to investigate internal consistency. The Cronbach’s Alpha for the SVENIS questionnaire is 0.95, which was an acceptable value [30].

Considering the participants worked in different residences and had different years of experiences (as shown in Table 1), we conducted inter-group comparison to check whether there are significant differences in the perceptions of participants in terms of their experiences of person-centred care in nursing homes.

A Kruskal-Wallis test was used to perform the inter-group comparison in this study. The test is a rank-based non-parametric statistical test that has been widely used to check the potential differences among three or more different groups. The suggested minimum sample size for a Kruskal-Wallis test is 45 responses per group [31] and the responses per area were more than 45 responses (Table 1). Multiple regression analyses were then used to explore the variables that predict the total SVENIS score. The suggested minimum sample size for a multiple standard regression is 25 responses per variable [32] and hence the responses were enough for the analysis (Table 1). Using a standard multiple regression, the following variables were entered in the model: total SVENIS score, sex, age, education, years of working in elderly care, years of working in the current nursing home, types of employment and the participant’s current nursing home. All analyses were conducted using the SPSS version 27 and the values of *p* < 0.05 were considered statistically significant. Missing data were excluded from the analyses depending on the comparisons that were analysed.

## Results

### Demographic participant characteristics

In total, 471 SVENIS questionnaires were answered, of which 463 were answered by NS and 8 by the FLMs from 9 NHs (Table 1). The eight FLMs have education as registered nurse, assistant nurse, and occupational therapist. Most of the participants were females (89.2%) and over than half of them were age 46 or over (55.4%). The majority of the NAs had healthcare education (82.2%), including education in care assistant and nurse assistant. Over half of them had been working within elderly care for over 16 years (55.8%). Around two-thirds of the participants were employed as staff for day and evening shifts (74.4%) and a high percentage of the staff were currently working in permanent NHs (66.2%).

**Table 1:** Demographic characteristics of the participants

Characteristics	Categories	N	%
Sex	Female Male Missing	422 28 21	89.6 5.90 4.50
Age	25 or under 26–45 46 or over Missing	27 137 262 47	5.70 29.1 55.6 9.60
Education	No healthcare education Nurse Assistant^a^ Registered nurse/occupational therapist Care assistant^b^ Missing	4 389 3 61 14	0.80 82.6 0.60 13.0 3.00
Years of working with elderly care	1 year or under 2 to 5 years 6–15 years 16 years or over Missing	20 56 103 264 28	4.20 11.9 21.9 56.1 5.90
Years of working in the current nursing home	1 year or under 2 to 5 years 6–15 years 16 years or over Missing	54 141 172 40 64	11.5 29.9 36.5 8.50 13.6
Type of employment	Day shift only Day/evening shift Night shift only Day/evening/night Others Missing	10 352 58 28 2 21	2.10 74.7 12.3 5.90 0.40 4.50
The participant’s current nursing home type	Permanent residence for persons with dementia Permanent elderly residence Service residence Temporary residence Missing	114 313 0 32 12	24.2 66.5 0.00 6.80 2.50

^a^Nurse assistant is a 3-year education in an upper secondary school, i.e., not a university degree

^b^Care assistant is an education around 30 weeks

### Inter-group comparisons

According to the Kruskal-Wallis test results (Table 2), no significant differences were found in *sex, age groups, education, years of working in elderly care* and *years of working in their current elderly residence*.

Significant differences were found among *different types of employment* and among *current nursing home type*. In terms of different types of employment, the participants who only work night shifts scored significantly lower in the total SVENIS score, area 2-work climate, area 3-person-centered care, and area 4-work condition than the other groups.

The participants who worked in *permanent nursing homes for dementia* scored significantly higher in the total SVENIS score and in all the individual areas in the SVENIS questionnaire. Further individual analyses of the 24 questions in the area of leadership showed that all questions were scored significantly higher (*p* < 0.05) among the staff who worked in permanent nursing homes for dementia (data not shown).

**Table 2:** Comparisons of the five areas in SVENIS

Characteristics	Total	Area 1: Perceived organizational support	Area 2: Work climate	Area 3: Person-centred care	Area 4: Work condition	Area 5: Leadership
Mean rank (Median, Range)						
Sex						
Male	231.36 (314, 245–351)	201.29 (27, 14–39)	224.71 (61, 33–70)	224.98 (47, 34–60)	225.83(78, 10–89)	224.86 (94, 6-120)
Female	225.11 (311, 146–394)	227.11 (29, 13–45)	237.38 (62, 20–70)	233.27(47, 15–60)	220.55 (78, 10–96)	235.13 (98, 49–120)
*p*-value	0.806	0.308	0.617	0.744	0.835	0.686
Age						
25 or under	204.93 (312, 246–366)	196.83 (27, 18–39)	234.81 (63, 47–70)	240.22 (49, 32–60)	170.26 (75, 60–85)	198.48 (96, 49–118)
26–45	219.10 (314, 117–374)	209.85 (29, 14–44)	226.58 (62, 30–70)	218.34 (46, 24–60)	212.95 (79, 51–93)	216.80 (95, 26–120)
46 or over	211.46 (311, 180–394)	217.12 (29, 13–45)	204.46 (60, 27–70)	208.21 (47, 18–60)	218.25 (78, 10–96)	213.32 (94, 6-120)
*p*-value	0.784	0.655	0.151	0.373	0.155	0.778
Education						
No healthcare education	252.75 (315, 278–439)	194.25 (28, 18–34)	246.63 (61, 60–63)	214.63 (46, 42–49)	186.50 (75, 68–82)	294.25 (106, 75–118)
Nurse Assistant	224.88 (310, 146–394)	228.68 (29, 13–45)	222.72 (60, 20–70)	226.07 (46, 15–60)	227.57 (77, 10–96)	225.79 (93, 6-120)
Registered nurse	277.50 (312, 302–314)	294.75 (31, 29–33)	159.50 (55, 47–63)	224.25 (46, 41–51)	297.75 (83, 73–93)	298.25 (102, 98–108)
Care assistant	248.40 (320, 117–368)	227.42 (28, 18–42)	266.42 (63, 31–70)	245.02 (48, 24–60)	234.93 (78, 57–94)	239.17 (96, 26–120)
*p-*value	0.552	0.853	0.092	0.768	0.771	0.548
Years of working in elderly care						
1 year or under	260.02 (319, 247–368)	207.95 (28, 18–39)	273.75 (64, 41–70)	268.88 (48, 39–60)	204.03 (76, 60–93)	262.70 (100, 59–120)
2 to 5 years	232.26 (315, 177–374)	215.45 (29, 20–41)	233.24 (63, 32–70)	230.44 (46, 24–58)	227.50 (78, 51–93)	227.91 (97, 12–120)
6–15 years	229.06 (314, 209–374)	237.29 (29, 18–44)	229.26 (60, 33–70)	234.55 (47, 25–60)	217.04 (77, 57–94)	222.23 (94, 36–120)
16 years or over	214.19 (308, 146–394)	218.49 (29, 13–45)	212.86 (60, 20–70)	211.76 (46, 15–60)	224.13 (78, 10–96)	217.57(94, 6-120)
*p*-value	0.331	0.559	0.146	0.132	0.867	0.483
Years of working in the current nursing home						
1 year or under	211.48 (313, 218–366)	182.01 (28, 18–42)	217.73 (61, 43–70)	225.35 (48, 25–60)	179.14 (76, 51–87)	218.74 (97, 31–120)
2 to 5 years	213.42 (314, 177–374)	209.36 (29, 16–44)	211.71 (61, 27–70)	208.26 (46, 24–60)	210.85 (78, 57–92)	210.90 (96, 12–120)
6–15 years	194.31 (307, 192–384)	201.43 (28, 13–42)	195.67 (60, 30–70)	193.81(47, 15–60)	204.04 (78, 10–94)	196.67 (91, 6-120)
16 years or over	202.38 (305, 201–371)	225.84 (30, 19–45)	194.11 (61, 31–70)	203.99 (46, 35–60)	213.24 (78, 55–90)	191.28 (89, 32–120)
*p*-value	0.513	0.301	0.477	0.353	0.370	0.481
Types of employment						
Day shift only	296.65 (329, 242–369)	167.00 (26, 13–37)	283.00 (64, 52–70)	228.15 (46, 37–58)	300.75 (84, 65–92)	312.80 (107, 60–120)
Day/evening shift	226.51 (313, 117–394)	227.24 (29, 14–45)	226.88 (61, 27–103)	233.95 (48, 18–60)	224.61 (78, 10–96)	223.53 (94, 6-120)
Night shift only	180.10 (298, 156–366)	202.25 (28, 16–41)	176.27 (58, 30–70)	152.28 (40, 18–60)	191.66 (74, 53–94)	202.85 (88, 21–120)
Day/evening/night	265.41 (319, 209–374)	256.63 (30, 25–39)	273.54 (65, 34–70)	254.04 (49, 30–60)	263.89 (79, 63–93)	249.98 (97, 30–120)
*p*-value	0.005*	0.133	0.003*	0.000*	0.020*	0.061
The participants’ current nursing home type						
Permanent home for persons with dementia	249.57 (316, 201–371)	253.15 (30, 14–44)	237.14 (62, 27–70)	241.51 (48, 20–60)	243.72 (79, 55–93)	252.48 (99, 39–120)
Permanent nursing home for older adults	234.92 (312, 177–394)	228.35 (28, 16–43)	232.58 (60, 32–70)	225.57 (46, 15–60)	232.18 (78, 10–96)	233.53 (94, 21–120)
Service residence	-	-	-	-	-	-
Temporary residence	112.17 (257, 177–351)	163.72 (27, 13–38)	150.95 (56, 20–67)	133.14 (38, 18–60)	159.77 (72, 56–96)	115.42 (60, 6-120)
*p*-value	0.000*	0.003*	0.002*	0.000*	0.006*	0.000*

*p value ≤ 0.05 *is considered as statistically significant*

### Predictors of SVENIS score

In the standard multiple regression, the participants’ current NH was the only variable that made a significant contribution to the prediction of the SVENIS total score (Table 3). The participants’ current nursing home explained 24.4 percent of the variance in the SVENIS score.

**Table 3:** Standard multiple regression with Svenis total score as the dependent variable

Variables	Standardized β	*t*	*p*	95% Confidence Intervals for β	
				Lower Bound	Upper Bound
Sex	-0.058	-1.113	0.266	-22.351	6.196
Age	0.054	0.833	0.405	-4.718	11.652
Education	-0.017	-0.324	0.746	-7.088	5.084
Years of working in elderly care	-0.103	-1.425	0.155	-11.691	1.865
Years of working in this nursing home	-0.011	-0.182	0.856	-6.162	5.121
Types of employment	0.012	0.226	0.821	-6.318	7.960
The participants’ current nursing home type	-0.244	-4.768	0.000*	-25.439	-10.582

*p value ≤ 0.05 *is considered as statistically significant*

## Discussion

The aim of the study was to evaluate NS and first-line managers’ perceptions of the opportunities to perform person-centred care during the pandemic. The results from the comparison analyses show that the staff from *permanent homes for persons with dementia* had the greater number of opportunities to perform person-centred care during the pandemic. The day shift staff had more opportunities to perform person-centred care than night shift staffs. The results from the standard multiple regression show that the respondent’s current nursing home was the most significant variable affecting the opportunities to perform PCC. The analyses of both the comparison analyses and the regression suggest that day shift staff from permanent homes for persons with dementia had greater number opportunities to perform person-centred care during the pandemic. The same group also rated the importance of leadership as being high for performing PCC.

Even though the experience of the COVID-19 has highlighted longstanding gaps in elderly care nationally and globally [33, 34], and care in NHs has been negatively reported [3, 35], the day shift staff in dementia care rated high the performance of PCC in NHs. Practice of PCC in dementia case is about ‘knowing the person’ and seeing the ‘whole’ person. It is advantageous to see older people as humans with a rich life history, not just as people with dementia who are time consuming and difficult to provide care. Studies have shown that PCC has been beneficially associated to older people health and well-being and associated with higher quality of life among persons with dementia [36, 37].

In Sweden, investments have been made in training staff caring for older persons with dementia diseases in these issues [17]. However, the staff are often recruited based on their special interest in working with people with dementia and the units they will work in are designed to be smaller. The staff included in the study had a long experience working in NHs. Altogether this may be an explanation for the higher ratings from NS in NHs for people with dementia. A previous study shows that staff are more likely to conduct PCC if the staff is involved in developing the individual care plan [35]. Another important factor for practicing PCC is clinical training practice [38]. This study describes the importance of being able to interact with the family when planning PCC. During the time of this study this may have been problematic because of the COVID restrictions, although it may have occurred to some extent in telephone conversations.

The night staff is the group that rates lowest in all areas in the questionnaire. Several factors may explain this. It may be due to the fact that they see the focus of their work as being the patient getting a good night’s sleep [39]. They also state that there is a lack of understanding from day staff about their work situation, which they consider to be unique. For example, they must work in silence, in poor lighting and when they are tired. A special relationship develops between RN and NS as they are in a dependent relationship with each other [39]. Night staff often request a more collaboration with day staff and managers [40] which may be a sign that leadership is valued lower than in other groups.

Several studies pointed out the importance of leadership in elderly care to set the standard of the care [20–22]. Day shift NS from permanent homes for persons with dementia within this study rated high the importance of leadership for performing PCC. This is in line with Backman’s findings regarding the importance of leadership in dementia homes [15]. Backman suggests that leadership is significantly associated with optimal PCC provision as well as with the work situation of staff. A highly rated leadership behaviour in NH is characterized by experimenting with new ideas, controlling work closely, relying on the subordinates, giving direct feedback, and handling conflicts constructively.

The fact that there are obvious challenges in providing PCC under social distance when there might be restrictions in interacting with patients, should encourage research and development into ways of enhancing PCC under such circumstances. Given the evolving nature of the dynamics of interaction and communication between patients and healthcare workers in the digital era, there is scope for research into novel strategies and techniques for interacting with patients. A greater awareness, through nurse related education of novel strategies for providing PCC would be of obvious benefit in the case of future pandemics. Research into different forms of practicing PCC is ongoing in developing countries [41]. One implication of this study for other countries with less supportive social welfare systems is to build a strong leadership for PCC implementation. Free courses can be provided to managers of NH to equip the managers with the right skills to implement PCC.

### Strength and limitations

Using the SVENIS questionnaire proved to be adequate as the instrument highlights the areas relevant for PCC as suggested by McCormack and McCance [18]. Some areas are semantically close to each other, for example, *Perceived organizational support* and *Work conditions*, which can mean that they might give similar scores in both areas, which could be a weakness of the study. Another possible weakness is the uncertainty whether self-reported data truly report the situations. There is a large number of questions in the questionnaire, and this may be a reason why only 471 of 711 NS or FLM participated. Another explanation for the low participation rate may have been the extraordinary situation that COVID-19 meant there was a high sick leave rate among the staff and stressful work situations.

One limitation was the percentage of missing data. Similar to other cohort studies [42], we chose to exclude participants with missing data and performed a complete-case analysis. This might affect the findings of this study. The impact of missing data could be substantial if the percentage of missing data was 50% or higher [43] and the percentage of missing data in our sample ranged from 2.5 to 13.6%. This suggests that the missing data in our sample did not have substantial effect on our findings. Another limitation was the influence of cofounding factors that existed among the residents. During the restrictions, the behaviour of the residents might not be the same as before the pandemic. The new behaviour could facilitate or be a hinder for PCC practice.

## Conclusions

Despite the restrictions in connection with COVID-19 and all the criticism directed against the care of older people, the day staff felt that they did in fact conduct PCC. Staff in nursing homes for dementia had the highest opportunities which may be because they are better prepared to engage with the individual. This is something that might be desirable for all staff in elderly care to receive training in. All staff would need the same conditions as staff in dementia homes to be able to perform PCC to the same degree. Overall, delivering PCC could be enhanced by taking into account societal changes and the fact that globally, the nature of interactions between patients and caregivers is changing. Intentions to adapt and enhance PCC to changing social circumstances must be informed by research which takes such changes into account. Nursing education must also reflect societal changes in how individuals in society interact and communicate with each other. This is also in line with the government’s trust reform, which aims for the public sector to have governance based on trust and confidence in employees’ experience and knowledge. The importance of leadership was also evident, which means that an investment in first-line managers is seen as necessary to conduct PCC. The WHO has just recently announced COVID-19 is no longer a public health emergency of international concern. Countries around the world have learnt the lessons of COVID. The results of this study can contribute to the preparation for future pandemics.

## Data Availability

The datasets used and/or analysed during the current study are available from the corresponding author upon reasonable request.

## Abbreviations

FLMs: First-line managers
NHs: Nursing homes
NS: Nurse staff
PCC: Person-centred care
RN: Registered Nurse
